# Do pneumococcal conjugate vaccines provide any cross-protection against serotype 19A?

**DOI:** 10.1186/1471-2431-10-4

**Published:** 2010-02-02

**Authors:** William P Hausdorff, Bernard Hoet, Lode Schuerman

**Affiliations:** 1 GlaxoSmithKline Biologicals, Rixensart, Belgium

## Abstract

**Background:**

Introduction of the 7-valent pneumococcal conjugate vaccine (7vCRM) in several countries has led to a rapid, significant drop in vaccine-type invasive pneumococcal disease (IPD) in immunized children. In the United States and some other countries with high antibiotic use, a subsequent rise in serotype 19A IPD has been taken to indicate that the 19F conjugate in the vaccine provides no cross-protection against the immunologically related 19A.

**Discussion:**

We systematically assessed the clinical efficacy and effectiveness of 19F-containing vaccines against 19A disease or nasopharyngeal carriage by searching English-language articles in the electronic databases PubMed, Current contents, Scopus, and Embase from 1985 to 2008. The vaccine efficacy and effectiveness point estimates were consistently positive for modest protection against 19A IPD and acute otitis media (AOM). However, statistical significance was not reached in any individual study. No consistent impact of 7vCRM on 19A nasopharyngeal colonization could be detected. These findings are discussed in context of immunogenicity analyses indicating that 7vCRM induces functionally active anti-19A antibodies after the booster dose, and that other 19F-containing vaccine formulations may elicit higher levels of such antibodies after both primary and booster doses.

**Summary:**

Taken together, these results suggest that 19F-conjugates can provide some protection against 19A disease. The magnitude of this protection in a given setting will likely depend on several factors. These include the anti-19A immunogenicity of the specific vaccine formulation, the number of doses of that formulation needed to elicit the response, and the burden of 19A disease that occurs after those doses. It is possible that a modest protective effect may be obscured by the presence of countervailing selection pressures (such as high antibiotic use) that favor an increase in colonization with antibiotic-non-susceptible strains of 19A.

## Background

Since the 7-valent pneumococcal conjugate vaccine (7vCRM; *Prevenar*™/*Prevnar*™; Wyeth Vaccines) was first introduced in the United States (US) in 2000, invasive pneumococcal disease (IPD) due to *Streptococcus pneumoniae *vaccine-serotypes (4, 6B, 9V, 14, 18C, 19F, and 23F) has dramatically decreased among children and adults [1-6].

Serotypes 6A and 19A were not included in 7vCRM and several other pneumococcal conjugate vaccines (PCVs) because it was anticipated that the immunological similarities of vaccine serotypes 6B and 19F, respectively, might elicit sufficient cross-protection [7]. There is now convincing evidence that the 6B conjugate contained in 7vCRM (in which the 6B polysaccharide is conjugated to a non-toxic variant of diphtheria toxin) provides a high degree of cross-protection against 6A IPD [8,9], acute otitis media (AOM) [10], and even offers a significant level of herd protection [8]. An efficacy trial with the 11Pn-PD vaccine candidate (in which 6B was conjugated to the non-typeable *Haemophilus influenzae *protein D) also suggested cross-protection against 6A AOM [11].

For serotype 19A, some preclinical studies support the possibility of cross-protection. For instance, serum from infants immunized with a 19F tetanus toxoid conjugate provided protection against lung infection caused by both 19F and 19A in a murine model [12]. Nonetheless, clear clinical evidence of cross-protection against 19A disease by 19F-containing PCVs has been lacking. Indeed, the rise in 19A IPD in certain populations well immunized with 7vCRM would seem to argue against it [1-6]. In the US, for example, the incidence of 19A IPD rose in children >5 from approximately 2.5 cases/100,000 in 1998-9 (prior to 7vCRM) to 9 cases/100,000 in 2005 [5].

However, analysis of clinical and post-licensure studies with 7vCRM and other pneumococcal conjugate vaccines suggests a more complex situation. This review discusses the literature on cross-protection against 19A disease and colonization provided by 19F-containing vaccines, and relates these results to recently published immunogenicity data.

## Discussion

### Clinical efficacy and effectiveness data

**Figure 1:**
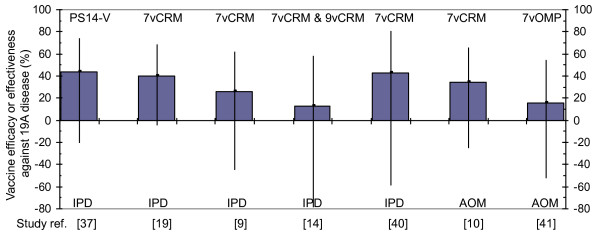
**Vaccine efficacy or effectiveness of 19F-containing vaccines against 19A disease**. Depicted are point estimates with 95% confidence intervals from individual studies or metanalyses with at least five 19A cases. PS14-V, 14 valent polysaccharide vaccine; 7vCRM, 7-valent conjugate vaccine with crm197 carrier; 9vCRM, 9-valent conjugate vaccine candidate with crm197 carrier; 7vOMP, 7-valent conjugate vaccine candidate with outer membrane protein carrier; IPD, invasive pneumococcal disease; AOM, acute otitis media. See Table 1 for more study details.

Table 1 presents a review of prospective and retrospective clinical studies and post-licensure surveillance studies with 7vCRM and other 19F-containing vaccines. We wish to highlight two findings. First, in every analysis with 5 or more 19A cases (Figure 1), the anti-19A point estimate is positive, with values ranging from 13% to 67%. This suggests that the true efficacy or effectiveness is probably not null. Secondly, however, none of the anti-19A efficacy or effectiveness point estimates is, by itself, statistically significant.

**Table 1:** Studies examining efficacy or effectiveness of 19F-containing vaccines against 19A IPD and AOM

Study or Analysis	Vaccine	Vaccine regimen	Endpoint	Age group	Total Number of Subjects	Numbers of 19F Cases	% VE against 19F (95% CI)	Numbers of 19A Cases	% VE against 19A (95% CI)
Polysaccharide vaccine			IPD						

US indirect cohort analysis [37]	PS14-V*	1 dose as per US recomm.	IPD	> 5 y	2837 total cases	12 (vacc) 32(unvacc)	**11** (-86,,56)	10 (vacc) 42(unvacc)	**44** (-21, 74)

									

Conjugate vaccines			IPD						

N. California Kaiser Permanente efficacy [15,38]^a^	7vCRM	2,4,6,12-15 mo	IPD after ≥ 3 doses	2 mo to ~3.5 y	18927 (vacc) 18941 (ctrl)	2 (vacc) 13 (ctrl)	**85** (32, 97)	0 (vacc) 1 (ctrl)	**67** (-719, 99)

Native Americans efficacy [16,38]^a^	7vCRM	2,4,6,12-15 mo	IPD after ≥ 3 doses	2 mo to 2 y	7532 (vacc) 7151 (ctrl)	0 (vacc) 1 (ctrl)	**68** (-677, 99)	1 (vacc) 0(ctrl)	**-185** (-900, 88)

South African HIV- efficacy [17,38]^a^	9vCRM	6, 10, 14 wks	IPD after 3 doses	6 wk to 6.2 y	18633 (vacc) 18626 (ctrl)	0 (vacc) 1 (ctrl)	**67** (-718, 99)	3 (vacc) 1 (ctrl)	**-200** (-900, 69)

South African HIV+ efficacy [17,38]^a^	9vCRM	6, 10, 14 wks	IPD after 3 doses	6 wk to 6.2 y	1289 (vacc) 1288 (ctrl)	5 (vacc) 3 (ctrl)	**-67** (-595,60)	3 (vacc) 6 (ctrl)	**50** (-99, 88)

Gambia efficacy [14,39]^a^	9vCRM	6, 10, 14 wks	IPD after 3 doses	6 wk to 2.9 y	8718 (vacc) 8719 (ctrl)	0 (vacc) 2 (ctrl)	**80** (-317, 99)	6 (vacc) 7 (ctrl)	**14** (-155, 71)

*Meta-analysis of preceding efficacy studies*(^*a*^)	*7vCRM & 9vCRM*	*see individual studies*			*55099 (vacc)* *54725 (ctrl)*	*7 (vacc)* *20 (ctrl)*	***61*** *(13, 82)*	*13 (vacc)* *15 (ctrl)*	***13*** *(-79, 58)*

US post-marketing surveillance [19]	7vCRM	2,4,6,12-15 mo; catch-up schedule for < 2 y	IPD (2^nd ^yr after launch)	> 2 y (not all vacc)	~433,000	79 (pre) 14 (post)	**83** (72, 90) (% decrease decrease in estimated rate from pre-7vCRM)	20 (pre) 12 (post)	**40** (-5, 68) (% decrease in estimated rate from pre-7vCRM)

US CDC case-control [9]	7vCRM	2,4,6,12-15 mo	IPD ≥ 1 dose	3 to 59 mo	n.a.	34 discordant sets	**87** (65, 95)	46 discordant sets	**26** (-45, 62)

Canada (Québec) [40]: case-control	7vCRM	2,4,12 mo	IPD ≥ 1 dose	<5 y	n.a.	3 (vacc); 8 (unvacc)	**93 **(61,99)	29 (vacc); 8 (unvacc)	**42** (-76, 79)

									

Conjugate Vaccines			AOM						

Finland [10]: efficacy (FinOM)	7vCRM	2,4,6,12 mo	AOM after ≥ 3 doses	6.5 to 24 mo	831 (vacc) 831 (ctrl)	43 (vacc) 58 (ctrl)	**25** (-14, 51)	17 (vacc) 26 (ctrl)	**34** (-26, 65)

Finland [41]: efficacy(FinOM)	7vOMP	2,4,6,12 mo	AOM after ≥ 3 doses	6.5 to 24 mo	831 (vacc) 831 (ctrl)	37 (vacc) 58 (ctrl)	**37** (1, 59)	22 (vacc) 26 (ctrl)	**16** (-53, 54)

Czech/Slovak [11]: efficacy (POET)	11Pn-PD	3,4,5,12-15 mo	AOM after ≥ 3 doses	5 to 27 mo	2489 (vacc) 2479 (ctrl)	24 (vacc) 43 (ctrl)	**44** (8, 66)	1 (vacc) 3 (ctrl)	**67** (-209, 97)

*PS14-V, 14 valent polysaccharide vaccine, contained 19F but not 19A; it was replaced in the early 1980s by the 23-valent vaccine containing both 19A and 19F serotypes. ^a^Each of the 5 analyses (from 4 separate studies) is also included in the metanalysis in italics. Mo, month; n.a., not available; VE, vaccine efficacy/effectiveness; wk, week; y, year; vacc, active vaccine group; unvacc, unvaccinated; ctrl, control; 7vCRM, 7-valent conjugate vaccine with CRM197 carrier; 9vCRM, 9-valent conjugate vaccine candidate with CRM197 carrier; 7vOMP, 7-valent conjugate vaccine candidate with outer membrane protein carrier; IPD, invasive pneumococcal disease; AOM, acute otitis media

There is considerable variability in the point estimates in Table 1, which could conceivably be related to the different numbers of vaccine doses received by children in each study. There is support for this concept for 6B and 6A. Dagan et al., showed that whereas three primary doses (at 2,4, and 6 months of age) of 7vCRM significantly decreased both 6B and 6A nasopharyngeal colonization as measured in the 2^nd ^year of life, two primary doses alone (4 and 6 months) decreased 6B but not 6A colonization [13].

Two of the studies in Table 1 may provide some insight here. First, the analysis with the lowest point estimate for 19A cross-protection (13% [95% confidence intervals, -79% to 58%]) is a weighted meta-analysis [14] of four randomized, double-blind, placebo-controlled, efficacy studies using 7vCRM [15,16] or the related 9vCRM vaccine [17,18]. Twenty six of the 28 19A cases included in this analysis were contributed by the two 9vCRM studies conducted in Africa in which no booster dose was given.

Also potentially supporting the importance of a booster dose is the heterogeneous post-marketing experience with 19A in the US. The original report on the impact of 7vCRM introduction in the US pointed to a 40% (p = 0.09) decrease in the incidence of serotype 19A IPD in children younger than 2 years of age between the pre-7vCRM years 1998-1999 and the first full post-7vCRM year 2001 [19]. This decrease contrasts with the rise in 19A disease detected starting in 2002 in the same active surveillance system [3] and which continued for several years [5]. The onset of the rise coincided with the severe shortage of 7vCRM in the US between 2001 and 2004 that resulted in many children receiving fewer than three primary doses and/or no booster dose [20].

In contrast to the IPD and AOM data summarized in Table 1, there does not appear to be evidence of a consistent 7vCRM effect on 19A colonization itself (Table 2), even among different analyses within the same vaccine study population [21-23]. In fact, in some 7vCRM carriage studies at certain ages or time points, an apparent *increase *in 19A carriage is observed (odds ratios greater than 1 or negative point estimates for efficacy) following vaccination, although these changes were generally not statistically significant [22]. This suggests that any effect of 7vCRM on 19A may be limited to prevention of disease once colonized, but not on preventing 19A colonization in the first place.

**Table 2:** Studies examining efficacy or effectiveness of 19F-containing vaccines against 19A carriage

Study & location	Vaccine	Vaccine regimen	Age group(s) sampled	Number of Subjects	Number of 19F isolates	Efficacy against 19F VE or OR (95% CI or p value)	Number of 19A isolates	Efficacy against 19A VE or OR (95% CI or p value)
South Africa [42]: double blind, randomized, efficacy	9vCRM	6,10,14 wk	9 mo	242 (vacc) 239 (ctrl)	19 (vacc) 32 (ctrl)	VE 42%* (p = 0.05)	7 (vacc) 11 (ctrl)	VE 37%* (NS)

Israel [43]: double blind, randomized efficacy	9vCRM	12-24 mo received 2 doses; ≥ 24 mo 1 dose	15-35 mo 36-≥ 48 mo	816 (vacc) 790 (ctrl) 1071 (vacc) 1073 (ctrl)	41 (vacc) 62 (ctrl) 57 (vacc) 52 (ctrl)	OR 0.58 (p = 0.08) OR 1.11 (p = 0.69)	n.a. = 113 total (all groups combined)	OR 0.60** (p = 0.38) OR 1.25** (p = 0.56)

US Navajo Apache [21]: double blind, randomized efficacy	7vCRM	2,4,6,12-15 mo (87%)	1-7 y (median 3.3y) All >12 mo since last dose	468 (vacc) 281 (ctrl)	24 (vacc) 16 (ctrl)	OR 0.89 (0.47-1.72)	12 (vacc) 10 (ctrl)	OR 0.71 (0.30-1.67)

US Navajo Apache [23]: double blind, randomized efficacy	7vCRM	2,4,6,12-15 mo	7 mo	227 (vacc) 226 (ctrl)	2 (vacc) 11 (ctrl)	OR not calculated because "small sample size resulted in non-convergence of model"	10 (vacc) 4 (ctrl)	OR 1.75 (0.41-7.58)
			12 mo	226 (vacc) 208 (ctrl)	7 (vacc) 8 (ctrl)		3 (vacc) 7 (ctrl)	OR 0.38 (0.09-1.57)
			18 mo	239 (vacc) 219 (ctrl)	18 (vacc) 8 (ctrl)		14 (vacc) 10 (ctrl)	OR 1.68 (0.7-4.05)

US Navajo Apache [22]: double blind, randomized efficacy	7vCRM	Infants: 2,4,6, 12-15 mo; 12-23 mo: 2 doses	Unvaccinated children/adults in study households	2048 (vacc) 1376 (ctrl)	29 (vacc) 37 (ctrl)	OR 0.52 (0.31-0.87)	15 (vacc) 23 (ctrl)	OR 0.34 (0.16-0.75)

France [44]: effectiveness	7vCRM	≥ 1 dose	Children w/AOM 6-24 mo (mean 14 mo)	575 (vacc) 1331 (ctrl)	59 (vacc) 178 (ctrl)	24%* (p = 0.06)	50 (vacc) 92 (ctrl)	-26%* (p = 0.2)

Greece [45]: effectiveness	7vCRM	12-23 mo: 2 doses (13%); ≥ 24 mo: 1 dose (85%)	13-76 mo (median 47 mo)	285 (vacc) 582 (ctrl)	24 (vacc) 64 (ctrl)	36%*	3 (vacc) 14 (ctrl)	58%*

*Only case numbers provided in original paper; approximate % VE calculated for this table;

**confidence intervals for odds rations not provided in original publication.

Mo, month; NS; not significant; OR, odds ratio; VE, vaccine efficacy/effectiveness; wk, week; y, year. 7vCRM, 7-valent conjugate vaccine with CRM197 carrier.  9vCRM; 9-valent conjugate vaccine candidate with CRM197 carrier.

### Immunogenicity data

The ability of pneumococcal vaccines to induce functional anti-capsular polysaccharide antibodies is considered to be the major mechanism by which these vaccines prevent IPD [24,25]. Vaccine-induced antibody levels measured by ELISA [25] and especially by opsonophagocytic activity assays (OPA) against individual pneumococcal serotypes have been shown to correlate well with clinical effectiveness [26]. Henckaerts et al. [24] showed that, following three primary doses of 7vCRM, virtually no child (3%) developed anti-19A OPA above the threshold suggested to correlate with clinical effectiveness against IPD (i.e., OPA titer ≥8) [25,26]. More recent studies from three different laboratories are consistent with these findings [27-29]. In contrast, a booster dose, of 7vCRM elicited some anti-19A OPA activity, with approximately 25%-30% of children having OPA titers ≥8 [29,30].

Other pneumococcal conjugate vaccines with distinct chemistries and carriers may have different immunological properties. For example, it has been observed that the periodate oxidation step used to link the 19F polysaccharide to the CRM_197 _carrier protein in 7vCRM prior to reductive amination alters the antigenicity of the polysaccharide [31]. In three separate studies [29,30,32], 20%-30% of children receiving a 3-dose primary series of PHiD-CV (a recently licensed 10-valent pneumococcal non-typeable *H. influenzae *protein Dconjugate vaccine in which 19F is conjugated to diphtheria toxoid; *Synflorix*™, GlaxoSmithKline Biologicals) had 19A OPA titers ≥8, compared with fewer than 5% of 7vCRM-immunized children in the same studies. Furthermore, a booster dose in the second year of life led to 19A OPA titers above this level in approximately 50% of PHiD-CV-immunized children and 30% of 7vCRM-immunized children [29,30].

Differences in OPA levels may reflect variability not only in the quantity, but also in the quality of the antibodies elicited by different conjugates. Nurkka et al. studied the functionality of cross-reactive antibodies induced by 7vCRM, 7vOMPC (polysaccharides conjugated to outer membrane proteins from *Neisseria meningitidis*, Merck Sharpe & Dohme), and 11Pn-PD, each with 19F conjugated to different carrier proteins [33]. In serum samples selected to have similar antibody concentrations as measured by ELISA, the 19A OPA titers were higher in the 11Pn-PD group than in the two other groups [33].

## Conclusion

The clinical efficacy, effectiveness, and immunogenicity studies summarized here suggest that immunization with 19F-containing pneumococcal conjugate vaccines can provide some direct protection against 19A disease. Nonetheless, the limited anti-19A activity of 7vCRM in particular, which appears insufficient to prevent 19A colonization, was evidently also insufficient to prevent or reverse [5] a net rise in 19A disease in young children in countries where high antimicrobial use has led to greatly increased colonization by antibiotic non-susceptible strains, including certain clones of 19A [5,34-36]. This does not exclude the possibility, however, that the modest effect of 7vCRM on disease may have attenuated or slowed down the rise. In any case, a protective effect of 7vCRM against 19A is likely to be most readily detectable in fully immunized children following a booster dose, and unfortunately, much of the IPD burden occurs prior to that time [19]. On the other hand, certain vaccines with different conjugation methods and carrier proteins have shown immunological cross-reactivity against 19A, even following a primary dose series, and could conceivably show greater protection against disease. Whether they offer any protection against 19A colonization remains to be determined. Accordingly, the potential for clinically significant cross-protection against 19A (and 6A) disease needs to be independently considered for each new vaccine.

## Methods

To systematically assess the clinical efficacy or effectiveness of 19F-containing vaccines against 19A disease or nasopharyngeal carriage, we searched for English-language articles in the electronic databases PubMed, Current contents, Scopus, and Embase from 1985 to 2008, with the latest search conducted 15 May 2009. The keywords used were "*S pneumoniae*" "19A", "19 A", "pneumococcal vaccine [MeSH Terms]", "carriage", "colonization", "invasive pneumococcal disease", and "acute otitis media." In addition, we also consulted recent reviews describing clinical trial results on nasopharyngeal carriage with 7vCRM for further references and, where possible, searched abstracts from major infectious diseases meetings in 2008 and the first half of 2009. For studies on disease, we only included reports in which the authors themselves provided vaccine efficacy or effectiveness calculations for 19A. The results are summarized in tabular format in lieu of a formal metanalysis, as the latter was considered inappropriate due to the large variability in study design (efficacy or effectiveness), vaccine dosing schedule, vaccines used, and disease endpoints (IPD, AOM). Because there were relatively few reports of vaccine effect on 19A nasopharyngeal carriage that included vaccine efficacy or effectiveness calculations, we also included studies where we could make those calculations based on case numbers.

## • Summary

• Since the introduction of the 7-valent pneumococcal conjugate vaccine (7vCRM), a rise in serotype 19A disease has been observed in the US and some other countries with high antibiotic use, and this has been taken to indicate that the 19F conjugate included in the vaccine does not provide any cross-protection against serotype 19A.

• However, clinical and post-licensure studies with several 19F-containing vaccines, including 7vCRM, consistently report modest clinical cross-protection against 19A disease in fully immunized individuals.

• Consistent with this finding, 7vCRM elicits anti-19A functional antibody responses that are detectable after the booster dose.

• New pneumococcal conjugate vaccines using different conjugation methods or carrier proteins which elicit earlier and/or higher functional anti-19A antibody responses could be more cross-protective against 19A disease than 7vCRM.

## Footnote

Prevenar/Prevnar is a trademark of Wyeth Vaccines.

Synflorix is a trademark of GlaxoSmithKline group of companies.

## Competing interests

**Financial support: **GlaxoSmithKline Biologicals paid for all costs associated with the development and the publishing of the present manuscript. The corresponding author had full access to the data and final responsibility for submission of the publication.

**Competing interests: **Drs. Hausdorff, Hoet and Schuerman declare they are employed and own stock in GlaxoSmithKline Biologicals, which has a licensed pneumococcal conjugate vaccine.

## Authors' contributions

WPH reviewed the literature and, together with BH and LS, analyzed the data and wrote the manuscript. All authors read and approved the final manuscript.

## Pre-publication history

The pre-publication history for this paper can be accessed here:

http://www.biomedcentral.com/1471-2431/10/4/prepub

## References

[B1] Pelton SI Huot H Finkelstein JA Bishop CJ Hsu KK Kellenberg J Huang SS Goldstein R Hanage WP Emergence of 19A as virulent and multidrug resistant Pneumococcus in Massachusetts following universal immunization of infants with pneumococcal conjugate vaccine Pediatr Infect Dis J 2007 26 6 468 472 10.1097/INF.0b013e31803df9ca 17529860

[B2] Singleton RJ Hennessy TW Bulkow LR Hammitt LL Zulz T Hurlburt DA Butler JC Rudolph K Parkinson A Invasive pneumococcal disease caused by nonvaccine serotypes among alaska native children with high levels of 7-valent pneumococcal conjugate vaccine coverage JAMA 2007 297 16 1784 1792 10.1001/jama.297.16.1784 17456820

[B3] Hicks LA Harrison LH Flannery B Hadler JL Schaffner W Craig AS Jackson D Thomas A Beall B Lynfield R Incidence of pneumococcal disease due to non-pneumococcal conjugate vaccine (PCV7) serotypes in the United States during the era of widespread PCV7 vaccination, 1998-2004 J Infect Dis 2007 196 9 1346 1354 10.1086/521626 17922399

[B4] Kyaw MH Lynfield R Schaffner W Craig AS Hadler J Reingold A Thomas AR Harrison LH Bennett NM Farley MM Effect of introduction of the pneumococcal conjugate vaccine on drug-resistant Streptococcus pneumoniae N Engl J Med 2006 354 14 1455 1463 10.1056/NEJMoa051642 16598044

[B5] Moore MR Gertz RE Woodbury RL Jr Barkocy-Gallagher GA Schaffner W Lexau C Gershman K Reingold A Farley M Harrison LH Population snapshot of emergent Streptococcus pneumoniae serotype 19A in the United States, 2005 J Infect Dis 2008 197 7 1016 1027 10.1086/528996 18419539

[B6] Pai R Moore MR Pilishvili T Gertz RE Whitney CG Beall B Postvaccine genetic structure of Streptococcus pneumoniae serotype 19A from children in the United States J Infect Dis 2005 192 11 1988 1995 10.1086/498043 16267772

[B7] Robbins JB Austrian R Lee CJ Rastogi SC Schiffman G Henrichsen J Makela PH Broome CV Facklam RR Tiesjema RH Considerations for formulating the second-generation pneumococcal capsular polysaccharide vaccine with emphasis on the cross-reactive types within groups J Infect Dis 1983 148 6 1136 1159 6361173 10.1093/infdis/148.6.1136

[B8] Park SY Moore MR Bruden DL Hyde TB Reasonover AL Harker-Jones M Rudolph KM Hurlburt DA Parks DJ Parkinson AJ Impact of conjugate vaccine on transmission of antimicrobial-resistant Streptococcus pneumoniae among Alaskan children Pediatr Infect Dis J 2008 27 4 335 340 10.1097/INF.0b013e318161434d 18316986

[B9] Whitney CG Pilishvili T Farley MM Schaffner W Craig AS Lynfield R Nyquist AC Gershman KA Vazquez M Bennett NM Effectiveness of seven-valent pneumococcal conjugate vaccine against invasive pneumococcal disease: a matched case-control study Lancet 2006 368 9546 1495 1502 10.1016/S0140-6736(06)69637-2 17071283

[B10] Eskola J Kilpi T Palmu A Jokinen J Haapakoski J Herva E Takala A Kayhty H Karma P Kohberger R Efficacy of a pneumococcal conjugate vaccine against acute otitis media N Engl J Med 2001 344 6 403 409 10.1056/NEJM200102083440602 11172176

[B11] Prymula R Peeters P Chrobok V Kriz P Novakova E Kaliskova E Kohl I Lommel P Poolman J Prieels JP Pneumococcal capsular polysaccharides conjugated to protein D for prevention of acute otitis media caused by both Streptococcus pneumoniae and non-typable Haemophilus influenzae: a randomised double-blind efficacy study Lancet 2006 367 9512 740 748 10.1016/S0140-6736(06)68304-9 16517274

[B12] Jakobsen H Sigurdsson VD Sigurdardottir S Schulz D Jonsdottir I Pneumococcal serotype 19F conjugate vaccine induces cross-protective immunity to serotype 19A in a murine pneumococcal pneumonia model Infect Immun 2003 71 5 2956 2959 10.1128/IAI.71.5.2956-2959.2003 12704178 PMC153277

[B13] Dagan R Givon-Lavi N Janco J Greenberg D Nasopharyngeal Carriage of S. pneumoniae Vaccine Serotypes (VT-Sp) in First 2 Years of Life Following 4 Different 7-Valent CRM Conjugate Vaccine (PCV7) Schedules6th International Symposium on Pneumococci and Pneumoccocal Diseases: 8-12 June 2008 2008; Reykjavik, Iceland 2008

[B14] Klugman KP Cutts F Adegbola RA Black S Madhi SA O'Brien KL Santosham M Shinefield H Sterne JAC Siber GR, et al Meta-analysis of the efficacy of conjugate vaccines against invasive pneumococcal disease Pneumococcal Vaccines: the impact of conjugate vaccine 2008 Washington, DC.: ASM Press

[B15] Black S Shinefield H Fireman B Lewis E Ray P Hansen JR Elvin L Ensor KM Hackell J Siber G Efficacy, safety and immunogenicity of heptavalent pneumococcal conjugate vaccine in children. Northern California Kaiser Permanente Vaccine Study Center Group Pediatr Infect Dis J 2000 19 3 187 195 10.1097/00006454-200003000-00003 10749457

[B16] O'Brien KL Moulton LH Reid R Weatherholtz R Oski J Brown L Kumar G Parkinson A Hu D Hackell J Efficacy and safety of seven-valent conjugate pneumococcal vaccine in American Indian children: group randomised trial Lancet 2003 362 9381 355 361 10.1016/S0140-6736(03)14022-6 12907008

[B17] Madhi SA Adrian P Kuwanda L Jassat W Jones S Little T Soininen A Cutland C Klugman KP Long-term immunogenicity and efficacy of a 9-valent conjugate pneumococcal vaccine in human immunodeficient virus infected and non-infected children in the absence of a booster dose of vaccine Vaccine 2007 25 13 2451 2457 10.1016/j.vaccine.2006.09.019 17023095

[B18] Cutts FT Zaman SM Enwere G Jaffar S Levine OS Okoko JB Oluwalana C Vaughan A Obaro SK Leach A Efficacy of nine-valent pneumococcal conjugate vaccine against pneumonia and invasive pneumococcal disease in The Gambia: randomised, double-blind, placebo-controlled trial Lancet 2005 365 9465 1139 1146 10.1016/S0140-6736(05)71876-6 15794968

[B19] Whitney CG Farley MM Hadler J Harrison LH Bennett NM Lynfield R Reingold A Cieslak PR Pilishvili T Jackson D Decline in invasive pneumococcal disease after the introduction of protein-polysaccharide conjugate vaccine N Engl J Med 2003 348 18 1737 1746 10.1056/NEJMoa022823 12724479

[B20] CDC Notice to readers: Updated recommendations on the use of pneumococcal conjugate vaccine in a setting of vaccine shortage--Advisory Committee on Immunization Practices MMWR Morb Mortal Wkly Rep 2001 50 50 1140 1142

[B21] Millar EV O'Brien KL Watt JP Bronsdon MA Dallas J Whitney CG Reid R Santosham M Effect of community-wide conjugate pneumococcal vaccine use in infancy on nasopharyngeal carriage through 3 years of age: a cross-sectional study in a high-risk population Clin Infect Dis 2006 43 1 8 15 10.1086/504802 16758412

[B22] Millar EV Watt JP Bronsdon MA Dallas J Reid R Santosham M O'Brien KL Indirect effect of 7-valent pneumococcal conjugate vaccine on pneumococcal colonization among unvaccinated household members Clin Infect Dis 2008 47 8 989 996 10.1086/591966 18781875

[B23] O'Brien KL Millar EV Zell ER Bronsdon M Weatherholtz R Reid R Becenti J Kvamme S Whitney CG Santosham M Effect of pneumococcal conjugate vaccine on nasopharyngeal colonization among immunized and unimmunized children in a community-randomized trial J Infect Dis 2007 196 8 1211 1220 10.1086/521833 17955440

[B24] Henckaerts I Durant N De Grave D Schuerman L Poolman J Validation of a routine opsonophagocytosis assay to predict invasive pneumococcal disease efficacy of conjugate vaccine in children Vaccine 2007 25 13 2518 2527 10.1016/j.vaccine.2006.09.029 17034907

[B25] World Health Organization Recommendations for the production and control of pneumococcal conjugate vaccines WHO Technical Report Series. Annex 2 2005 927 Geneva, Switzerland: World Health Organization

[B26] Prymula R Schuerman L 10-valent pneumococcal nontypeable Haemophilus influenzae PD conjugate vaccine: Synflorix Expert Rev Vaccines 2009 8 11 1479 1500 10.1586/erv.09.113 19863240

[B27] Kieninger DM Kueper K Steul K Juergens C Ahlers N Baker S Giardina P Gruber W Scott D Safety and immunologic non-inferiority of 13-valent pneumococcal conjugate vaccine compared to 7-valent pneumococcal conjugate vaccine given as a 4-dose series with routine vaccines in healthy infants and toddlers48th Interscience Conference on Antimicrobial Agents & Chemotherapy (ICAAC). Washington DC, USA 2008

[B28] Lee H Nahm MH Burton R Kim KH Immune response in infants of the heptavalent pneumococcal conjugate vaccine against vaccine-related serotypes 6A and 19A Clin Vaccine Immunol 2009 10.1128/CVI.00344-08 PMC2650865 19144787

[B29] Vesikari T Wysocki J Chevallier B Karvonen A Czajka H Arsène J-P Lommel P Dieussaert I Schuerman L Immunogenicity of the 10-valent pneumococcal non-typeable *Haemophilus influenzae *Protein D Conjugate Vaccine (PHiD-CV) compared to the licensed 7vCRM vaccine Pediatr Infect Dis J 2009 28 4 Suppl S66 76 19325449 10.1097/INF.0b013e318199f8ef

[B30] Wysocki J Tejedor JC Grunert D Konior R Garcia-Sicilia J Knuf M Bernard L Dieussaert I Schuerman L Immunogenicity of the 10-valent pneumococcal non-typeable *Haemophilus influenzae *Protein D Conjugate Vaccine (PHiD-CV) when co-administered with different Neisseria meningitidis serogroup C conjugate vaccines Pediatr Infect Dis J 2009 28 4 Suppl S77 88 19325450 10.1097/INF.0b013e318199f609

[B31] Concepcion N Lee C-H Frasch CE Conjugation chemistry effects immunological reactivity and epitope expression of the pneumococcal 19F polysaccharide3rd International Symposium on Pneumococci and Pneumoccocal Diseases 2002 Anchorage, Alaska

[B32] Bermal N Szenborn L Chrobot A Alberto E Lommel P Gatchalian S Dieussaert I Schuerman L The 10-valent pneumococcal non-typeable *Haemophilus influenzae *Protein D conjugate vaccine (PHiD-CV) co-administered with DTPw-HBV/Hib and poliovirus vaccines: assessment of immunogenicity Pediatr Infect Dis J 2009 28 4 Suppl S89 96 19325451 10.1097/INF.0b013e318199f901

[B33] Nurkka A Lehtonen H Vuorela A Ekstrom N Kayhty H Functionality of antibodies against serotypes 6A and 19A induced by three different pneumococcal conjugate vaccines (PCV) in infants5th International Symposium on Pneumococci and Pneumoccocal Diseases: 2-6 April 2006 2006; Alice Springs, Australia 2006

[B34] Van Effelterre T Moore M Whitney C Fierens F Hausdorff W A Dynamic Transmission Model of Invasive Pneumococcal Disease (IPD): Implications for Serotype 19A48th Interscience Conference on Antimicrobial Agents & Chemotherapy (ICAAC). Washington DC, USA 2008

[B35] Dagan R Givon-Lavi N Leibovitz E Greenberg D Porat N Introduction and Proliferation of Multidrug-Resistant Streptococcus pneumoniae Serotype 19A Clones That Cause Acute Otitis Media in an Unvaccinated Population J Infect Dis 2009 199 6 776 85 10.1086/597044 19434927

[B36] Hwa Choi E Hee Kim S Wook Eun B Jung Kim S Hee Kim N Lee J Jong Lee H Streptococcus pneumoniae serotype 19A in children, South Korea Emerg Infect Dis 2008 14 2 275 281 10.3201/eid1402.070807 18258121 PMC2600206

[B37] Butler JC Breiman RF Campbell JF Lipman HB Broome CV Facklam RR Pneumococcal polysaccharide vaccine efficacy. An evaluation of current recommendations JAMA 1993 270 15 1826 1831 10.1001/jama.270.15.1826 8411526

[B38] Klugman KP Walsh AL Phiri A Molyneux EM Mortality in penicillin-resistant pneumococcal meningitis Pediatr Infect Dis J 2008 27 7 671 672 10.1097/INF.0b013e31817709cf 18520964

[B39] Saaka M Okoko BJ Kohberger RC Jaffar S Enwere G Biney EE Oluwalana C Vaughan A Zaman SM Asthon L Immunogenicity and serotype-specific efficacy of a 9-valent pneumococcal conjugate vaccine (PCV-9) determined during an efficacy trial in The Gambia Vaccine 2008 26 29-30 3719 3726 10.1016/j.vaccine.2008.04.066 18514974

[B40] Deceuninck G De Wals P De Serres G Boulianne N Effectiveness of pneumococcal conjugate vaccine using a 2+1 infant schedule in Quebec, Canada. Pediatr Infect Dis J 2010 in press 20125062 10.1097/INF.0b013e3181cffa2a

[B41] Kilpi T Ahman H Jokinen J Lankinen KS Palmu A Savolainen H Gronholm M Leinonen M Hovi T Eskola J Protective efficacy of a second pneumococcal conjugate vaccine against pneumococcal acute otitis media in infants and children: randomized, controlled trial of a 7-valent pneumococcal polysaccharide-meningococcal outer membrane protein complex conjugate vaccine in 1666 children Clin Infect Dis 2003 37 9 1155 1164 10.1086/378744 14557958

[B42] Mbelle N Huebner RE Wasas AD Kimura A Chang I Klugman KP Immunogenicity and impact on nasopharyngeal carriage of a nonavalent pneumococcal conjugate vaccine J Infect Dis 1999 180 4 1171 1176 10.1086/315009 10479145

[B43] Dagan R Givon-Lavi N Zamir O Sikuler-Cohen M Guy L Janco J Yagupsky P Fraser D Reduction of nasopharyngeal carriage of Streptococcus pneumoniae after administration of a 9-valent pneumococcal conjugate vaccine to toddlers attending day care centers J Infect Dis 2002 185 7 927 936 10.1086/339525 11920317

[B44] Cohen R Levy C de La Rocque F Gelbert N Wollner A Fritzell B Bonnet E Tetelboum R Varon E Impact of pneumococcal conjugate vaccine and of reduction of antibiotic use on nasopharyngeal carriage of nonsusceptible pneumococci in children with acute otitis media Pediatr Infect Dis J 2006 25 11 1001 1007 10.1097/01.inf.0000243163.85163.a8 17072121

[B45] Grivea IN Panagiotou M Tsantouli AG Syrogiannopoulos GA Impact of heptavalent pneumococcal conjugate vaccine on nasopharyngeal carriage of penicillin-resistant Streptococcus pneumoniae among day-care center attendees in central Greece Pediatr Infect Dis J 2008 27 6 519 525 10.1097/INF.0b013e318168d28a 18469733

